# The association between educational attainment and sports participation: gender differences and the moderating role of family-of-origin social class

**DOI:** 10.3389/fpubh.2026.1838383

**Published:** 2026-06-29

**Authors:** Lai Xi Liu, Rui Du

**Affiliations:** 1 Department of Physical Education, Liaoning University of Technology, Jinzhou, China; 2 School of Physical Education, Liaoning University of Science and Engineering, Jinzhou, China

**Keywords:** cultural capital, educational attainment, family-of-origin social class, intergenerational transmission, sports participation

## Abstract

**Background:**

Many studies explore how education and family-of-origin social class affect sports participation. However, whether returns to education in sports participation differ by individuals’ social class of origin has received less systematic attention.

**Methods:**

Using the competing theories of resource compensation and resource reinforcement, this study examines the links among family of origin social class, education, and sports participation with data from the Chinese General Social Survey (CGSS) and ordered logit models. It asks whether the effect of education on sports participation varies by family-of-origin social class and whether the moderating role of family-of-origin social class in the education–sports participation association differs by gender.

**Results:**

The results show: (1) In the full sample, findings support resource reinforcement theory: among individuals from higher family-of-origin social class, higher educational attainment has a stronger positive effect on adult sports participation; (2) In gender-stratified analyses, findings support resource compensation theory: compared to men, women—who face a double disadvantage of gender and social class—incur higher costs in achieving educational returns in the field of sports, but the returns are more significant; (3) Parental education is positively associated with offspring’s sports participation, suggesting possible intergenerational transmission, and maternal education shows stronger statistical support than paternal education.

**Conclusion:**

This study contributes to understanding health inequalities related to gender and family of origin social class and has implications for improving health equity among disadvantaged groups. It suggests policy directions for promoting sports participation by raising offspring’s educational attainment.

## 1 Introduction

Health is fundamental to individual well-being, social advancement, and national development. The formulation and implementation of sound health policies are critical to enhancing population health and achieving sustainable social and economic progress. With the continuous expansion of educational opportunities and the improvement of residents’ educational attainment, education has become an important factor influencing individuals’ sport participation behavior. Relevant research shows that individuals with higher educational attainment are more likely to recognize the health value of physical activity, acquire sport-related knowledge, and develop regular exercise habits (1). The relationship between educational attainment and sports participation is likely to vary across family-of-origin social class. The socioeconomic conditions of one’s family of origin determine early-life sports resources, health awareness and physical activity routines (2). Individuals with higher educational attainment tend to have stable occupations, higher incomes, broader social networks, this information further encourages them to participate in physical activities, either directly or indirectly. Individuals from higher-class families usually receive more sport support, parental encouragement, and access to sport training during childhood, which may strengthen the positive effect of education on sport participation in adulthood (3). To examine the effect of educational attainment on participation in physical activity and the moderating role of family-of-origin social class. Accordingly, this study addresses three questions: Does the effect of individual education on sports participation vary by family-of-origin social class? Under different family-of-origin social class backgrounds, does this effect align more with resource compensation theory or resource reinforcement theory? Does the effect of education on sports participation show gender differentiation and intergenerational variation?

## 2 Literature review

### 2.1 Education level and sports participation

Sport participation is a core component of a healthy lifestyle and plays an important role in promoting individual health. Existing studies suggest that education is closely associated with health-related behaviors, including physical activity and sport participation. Ross’s “learning efficacy hypothesis” argues that education may enhance individuals’ ability to acquire, process, and apply sport- and exercise-related information, thereby improving their capacity to make informed decisions about sport participation (4). In this sense, educational attainment may influence sport participation by increasing individuals’ knowledge of exercise, improving their understanding of the benefits and risks of physical activity, and facilitating the adoption of more regular and structured exercise behaviors. Studies of adults indicate pronounced educational disparities across various domains of physical activity, especially in leisure-time and sport-related activities, where individuals with higher education consistently exhibit greater participation (5). Similarly, research in Scotland finds that educational attainment is associated with both the likelihood of participating in sport and the duration of sport activities. Education may therefore promote sport participation by improving sport-related knowledge, increasing awareness of the importance of regular exercise, and enhancing access to supportive environments and resources for physical activity (6). Existing research mostly focuses on individuals’ educational attainment. There is limited attention to how parental education influences children’s sport participation. This is an important research gap, especially in China, where parents often decide children’s extracurricular activities, educational plans, and sport-related resources. Parental education may affect participation through parenting and resource allocation.

### 2.2 Family-of-origin social class and sports participation

At the early stages of the life course, the family constitutes the primary context of socialization and plays a fundamental role in shaping children’s physical and behavioral development through parenting practices, material provision, and the transmission of values (7). A growing body of research has shown that family-of-origin social class is closely associated with patterns of sports participation, with members of the same household often exhibiting similar participation preferences, frequencies, and attitudes. Such convergence suggests that sports behavior is not merely an individual choice, but is embedded in the family environment and shaped by intergenerational behavioral transmission (8). From the perspective of cumulative advantage and disadvantage, children from lower family-of-origin social class are more likely to be exposed to adverse life events and structural constraints. They also tend to have more limited access to educational resources, occupational mobility opportunities, and sport environments (9). Moreover, the social class of the family of origin may shape individuals’ sports participation across the life course by influencing the material conditions, parental support, and behavioral norms to which they are exposed in early life. In this regard, family-of-origin social class affects sports participation not only through the provision of resources but also through the transmission of attitudes, habits, and opportunities related to physical activity (10). Previous studies further indicate that family-of-origin social class and related internal family resources are important determinants of children’s sports participation. Such class-based disparities may lead to the accumulation of unequal opportunities and outcomes in sports participation over the life course (11). Given China’s distinctive family structure, social stratification, and patterns of sport participation, further research is needed to examine how family-of-origin social class shapes sports participation among Chinese populations.

### 2.3 Family-of-origin social class, educational level, input and return in sports participation

To clarify the theoretical framework guiding this study, we integrate resource compensation and resource reinforcement perspectives to explain how family-of-origin social class and education jointly shape sports participation. First, from a resource compensation perspective, education may play a stronger role among individuals from lower family-of-origin social class because it provides alternative resources—such as knowledge, skills, social networks, perceived control, and access to opportunities—that can offset disadvantages rooted in family-of-origin social class. In this case, higher educational attainment would narrow class-based gaps in sports participation. In contrast, the resource reinforcement perspective suggests that education and family-of-origin social class may accumulate advantages over the life course. Individuals from higher family-of-origin social class are more likely to convert educational attainment into sports participation because they possess additional family and community resources, including economic support, cultural familiarity with sport, and supportive social environments. Thus, in this scenario, education would reinforce rather than reduce inequalities in sports participation.

Ross and Mirowsky (12) argued that when individuals possess diverse resources, outcomes depend less on any single resource, whereas under resource scarcity, particular resources become more influential. This suggests that education may be especially important for individuals from lower family-of-origin social class. Consistent with this view, Andersson and Vaughan (13) found that the association between education and sports-related outcomes is stronger among less advantaged groups, indicating a possible compensatory role of education. Bauldry (14) indicated that education’s socio-economic benefits improve health outcomes among disadvantaged populations partly by increasing perceived control. Schafer et al. (15) emphasized that educational and family-of-origin social class may also reinforce each other across the life course, suggesting that individuals from higher family-of-origin social class may benefit more from education due to accumulated advantages. Therefore, this study examines whether education functions as a compensatory resource that promotes sports participation among individuals from lower family-of-origin social class, or as a reinforcing resource that amplifies existing family-of-origin social class inequalities family-of-origin social class may moderate the relationship between education and sports participation: individuals from advantaged social classes are better positioned to convert educational attainment into sports-related resources and opportunities, thereby accumulating further advantages and reinforcing existing patterns of social inequality (16). Family class advantage and educational attainment interact synergistically, and this synergy more strongly promotes individual sports participation (17). Drawing on prior argumentation and derivation, this paper therefore proposes two groups of competing theoretical hypotheses:

*Hypothesis 1a*: The lower the social class of the family of origin, the stronger the effect of education on sports participation.

*Hypothesis 1b*: The higher the social class of the family of origin, the stronger the effect of education on sports participation.

This gendered allocation of family resources suggests that gender may condition how family-of-origin social class moderates the relationship between education and sports participation. On the one hand, from the perspective of resource compensation, education may be especially important for females because it can help offset disadvantages caused by gender-biased family resource allocation (18). On this case, females from higher-class families may be better able to convert educational attainment into sports-related opportunities and participation, making the moderating effect of family-of-origin social class stronger among females. On the other hand, from the perspective of resource reinforcement, males may benefit more from the combined advantages of family class and education, because families tend to place greater emphasis on boys’ sports participation and are more likely to provide sons with financial, time, and social support for engaging in physical and extracurricular activities (19). In this case, the interaction between family-of-origin social class and education may be stronger among males. Based on these two competing mechanisms, the following hypotheses are proposed:

*Hypothesis 2a*: The moderating effect of family of origin social class on the relationship between education and sports participation is stronger among females than among males.

*Hypothesis 2b*: The moderating effect of family of origin social class on the relationship between education and sports participation is stronger among males than among females.

## 3 Methods

### 3.1 Data collection

The data in this study come from the 2021 Chinese General Social Survey (CGSS). The questionnaire comprises Module A (core), Module B (partial), and Module C (thematic), totaling approximately 700 variables. A multi-stage stratified probability proportional to size (PPS) sampling method was employed for nationwide data collection, resulting in 10,283 valid responses. The study examines the relationship between individuals’ subjective assessment of their childhood family’s socioeconomic status and their adult health. Respondents were therefore restricted to residents aged 18 years and above. Cases with missing values on income, parental education, and self-rated family of origin social class were excluded, resulting in a final analytical sample of 7,848.

To assess potential selection bias, we compared the included and excluded cases in terms of key demographic characteristics, main study variables, and covariates used in the analytical models. The results showed no statistically significant differences in most observed characteristics between the two groups, suggesting that case exclusion was unlikely to introduce substantial selection bias (Table 1).

**Table 1:** Comparison of included and excluded cases.

Variable	Included cases (*n* = 7,848)	Excluded cases (*n* = 2,435)	Test statistic	*p*-value
Dependent variable				
Sport participation				
Often	3,196(40.7%)	900 (36.9%)	*χ*^2^ = 0.12	0.79
Sometime	1,066(13.6%)	411 (16.8%)		
Rarely	898(11.4%)	295(12.1%)		
Never	2,688(34.3%)	829 (34.2%)		
Independent variable				
Education				
Primary school or below	2,473(31.5%)	731 (30.0%)	*χ*^2^ = 1.35	0.85
Junior high school	2,256(28.7%)	735 (30.1%)		
Senior high school/technical secondary school	1,463(18.6%)	433 (18.0%)		
College	620(7.9%)	212 (8.7%)		
Bachelor’s degree or above	1,036(13.3%)	324 (13.3%)		
Moderating variable				
Family-of-origin social class	4.2799(1.86)	4.27(1.88)	*t* = 0.35	0.73
Other variable				
Hukou				
Urban	3,146(40.0%)	902 (37.0%)	*χ*^2^ = 0.00	0.71
Age	51.25(17.50)	49.40(15.35)	*t* = 0.52	0.60
Income (log-transformed)	3.68(1.79)	3.66(1.81)	*t* = 0.81	0.02
Political identity				
Communist Party member	931(11.8%)	325 (13.3%)	*χ*^2^ = 0.01	0.82
Marital status				
Married	5,583(71.7%)	1,546 (63.4%)	*χ*^2^ = 0.02	0.89
Use Internet				
Internet usage	5,555(70.8%)	1,924 (79.0%)	*χ*^2^ = 0.00	0.65
Health status				
Healthy	4,219(53.7%)	1,090 (44.7%)	*χ*^2^ = 0.48	0.79
Fair	2,215(28.2%)	887 (36.4%)		
Unhealthy	1,414(18.1%)	458 (18.9%)		
Region				
Eastern region	2,830(36.1%)	820 (33.7%)	*χ*^2^ = 3.76	0.03
Central region	2,582(32.9%)	860 (35.3%)		
Western region	2086(26.5%)	605 (24.8%)		
Northeastern region	350(4.5%)	150 (6.2%)		

### 3.2 Measure of variables

#### 3.2.1 Dependent variable

The dependent variable, sports participation, was measured in terms of physical exercise. In the CGSS 2021 questionnaire, item A30 asks respondents, “Over the past year, what activities have you done in your free time?” Response options and codes were: “daily” = 5, “several times a week” = 4, “several times a month” = 3, “several times a year or less” = 2, “never” = 1. Descriptive statistics showed that respondents reporting “daily” participation made up less than 2% of the sample; to avoid instability when used as the reference category in regression models, responses were recoded as “frequent” (code 4) for “daily” or “several times a week,” “sometimes” (code 3) for “several times a month,” “rarely” (code 2) for “several times a year or less,” and “never” (code 1).

#### 3.2.2 Independent variable

Education was the independent variable, measured using CGSS 2021 item A7a (“highest level of education attained”). Educational attainment was coded from low to high as follows: 1 = primary school or less, 2 = junior secondary school, 3 = senior secondary school or technical secondary school, 4 = junior college, 5 = undergraduate degree or above.

#### 3.2.3 Regulated variable

Family of origin social class was measured by self-rated social status. This measure reflects both objective family-of-origin social class and individuals’ subjective perceptions. In CGSS 2021, item A43d asks, “When you were 14 years old, where would you place your family on the social ladder?” with a 1–10 scale, where 10 indicates the top, and 1 indicates the bottom. Scores of 1–3 were classified as low social class, scores of 4–7 as middle social class, and scores of 8–10 as high social class.

#### 3.2.4 Other variables

Other variables included hukou, sex, income, political affiliation, marital status, health status, and region. Hukou was coded as rural = 0, urban = 1. Sex was coded as female = 0, male = 1. Income was the natural logarithm of annual total income. Political affiliation was coded as non-Communist Party = 0, Communist Party member = 1. Marital status was coded as unmarried = 0, married = 1. Health status was coded as unhealthy = 0, fair = 1, healthy = 2. Region was coded as Northeast = 0, West = 1, Central = 2, East = 3 (20). These variables were included in the models to examine their associations with sports participation.

#### 3.2.5 Analytic procedure

To allow for heterogeneous causal effects across the ordered categories of the dependent variable, a generalized ordered Logit model was employed, specified as follows:


$$P(Y_{i}>s)=\frac{\exp(\alpha_{s}+X_{i}\beta_{s})}{1+\exp(\alpha_{s}+X_{i}\beta_{s})},s=1,\cdots ,m-1$$

To allow for heterogeneous causal effects across ordered categories of the dependent variable, a generalized ordered Logit model was used. In this model, *s* indicates a specific category of the ordered multinomial dependent variable “sports participation,” and *m* is the total number of categories. *X*_i_ represents the independent variables, such as education, family of origin social class, and income. *Y*_i_ is the ordinal position of the dependent variable. The coefficients *α*_s_ and *β*_s_ are estimated for each category.

All partial proportional odds (PPO) regression analyses were fitted in Stata. In the PPO specification, independent variable that satisfy the proportional odds assumption receive fixed parameters that do not vary across outcome categories; independent variable that violate the parallel-lines assumption are allowed to have category-specific parameters. After fitting the models, Wald tests were applied to the full model to assess overall conformity with the proportional odds assumption (21).

## 4 Results

### 4.1 Descriptive statistics

Among the 7,848 participants, females exhibited higher levels of sports participation than males. Regarding individual educational attainment, only the category of senior high school/technical secondary school had more males than females. The average social class of the family of origin was higher among females than among males. Descriptive statistics for these variables are presented in (Table 2).

**Table 2:** Descriptive statistics.

Variable	Total sample	Male	Female	*P*-value
Dependent variable				
Sport participation				
Often	3,196(40.7%)	1,526(42.7%)	1,670(39.0%)	
Sometime	1,066(13.6%)	502(14.1%)	564(13.2%)	0.000(0.034)
Rarely	898(11.4%)	408(11.4%)	490(11.5%)	
Never	2,688(34.3%)	1,136(31.8%)	1,552(36.3%)	
Independent variable				
Education				
Primary school or below	2,473(31.5%)	919(25.7%)	1,554(36.3%)	
Junior high school	2,256(28.7%)	1,082(30.3%)	1,174(27.5%)	
Senior high school/technical secondary school	1,463(18.6%)	776(21.7%)	687(16.1%)	0.000(0.443)
College	620(7.9%)	295(8.3%)	325(7.6%)	
Bachelor’s degree or above	1,036(13.3%)	500(14.0%)	536(12.5%)	
Moderating variable				
Family-of-origin social class	4.2799(1.86)	4.1999(1.87)	4.3468(1.84)	0.001(0.001)
Other variable				
Hukou				
Urban	3,146(40.0%)	1,501(42.0%)	1,645(38.4%)	0.047(0.428)
Age	51.25(17.50)	51.91(17.87)	50.69(17.17)	0.393(0.357)
Income (log-transformed)	3.68(1.79)	4.04(1.50)	3.37(1.95)	0.000(0.000)
Political identity				
Communist Party member	931(11.8%)	626(17.5%)	305(7.1%)	0.000(0.000)
Marital status				
Married	5,583(71.7%)	2,517(70.4%)	3,066(71.1%)	0.296(0.384)
Use Internet				
Internet usage	5,555(70.8%)	3,047(71.3%)	2,508(70.3%)	0.038(0.396)
Health status				
Healthy	4,219(53.7%)	1986(55.6%)	2,233(52.2%)	
Fair	2,215(28.2%)	988(27.7%)	1,227(28.7%)	0.000(0.005)
Unhealthy	1,414(18.1%)	598(16.7%)	816(19.1%)	
Region				
Eastern region	2,830(36.1%)	1,294(36.2%)	1,536(35.9%)	
Central region	2,582(32.9%)	1,142(31.9%)	1,440(33.7%)	
Western region	2086(26.5%)	965(27.1%)	1,121(26.2%)	0.533(0.660)
Northeastern region	350(4.5%)	171(4.8%)	179(4.2%)	
Sample size	7,848	3,572	4,276	

In the first three columns, values in parentheses represent percentages and standard deviations for each subgroup. In the last column, values in parentheses refer to homogeneity of variance tests. When Prob > 0.05 (*α* = 0.05), no significant deviation from the equal variance assumption is observed, indicating that the data support the homogeneity of variance assumption and the results are reliable.

### 4.2 Generalized ordered logit model analysis based on the full sample

**Figure 1:**
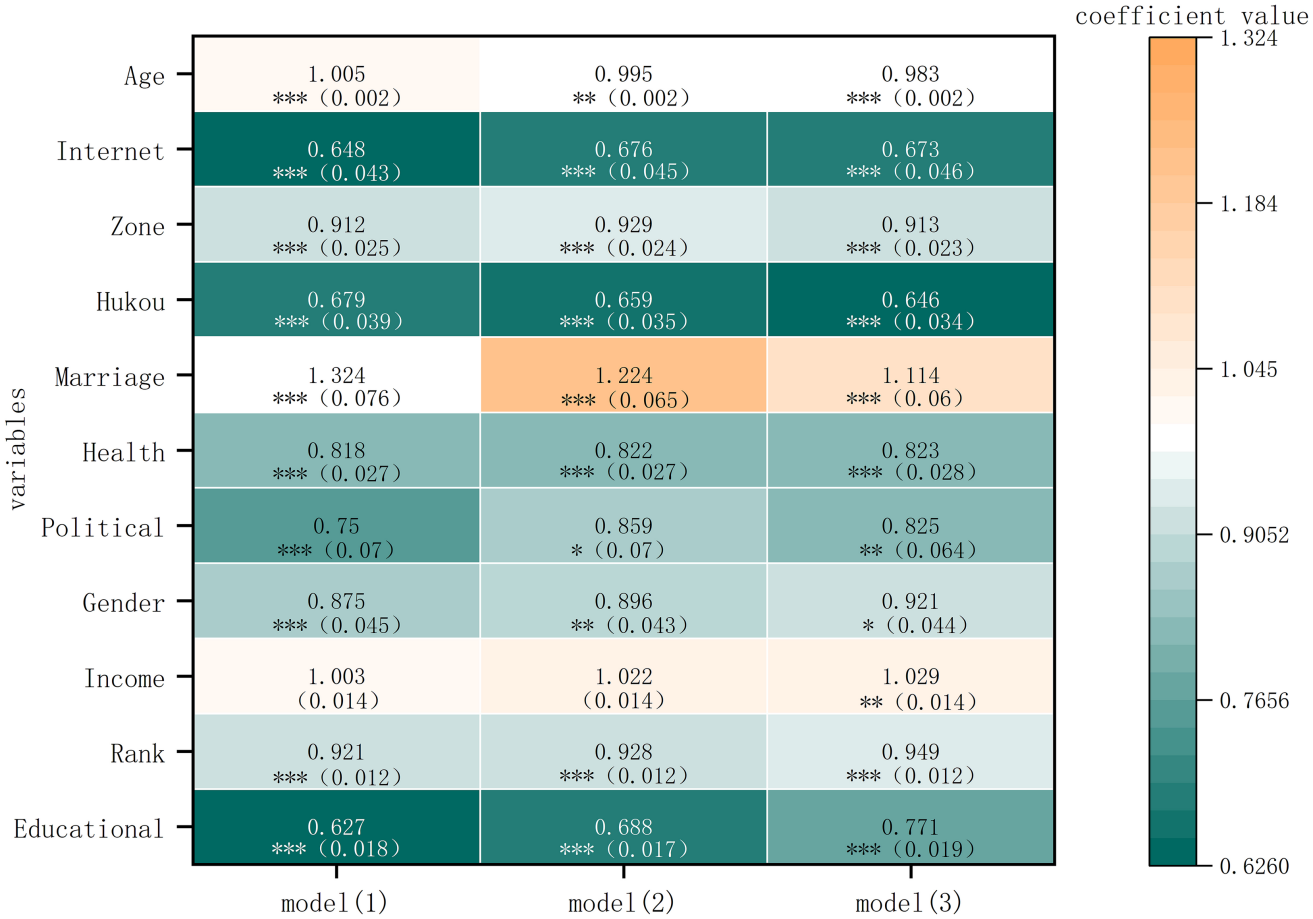
Visualization analysis of logit model.

The coefficient for the effect of education on sports participation was 0.627 (*p* < 0.001). After adding the education × family of origin social class interaction, the main effect of education remained significant (*β* = 0.673, *p* < 0.001), indicating that education is an important predictor of sports participation. Higher family social status was associated with more frequent sports participation (*β* = 0.944, *p* < 0.01; *β* = 0.934, *p* < 0.001; *β* = 0.937, *p* < 0.01). The education × family of origin social class interaction had a significant positive effect on sports participation (*β* = 0.983, *p* < 0.01; *β* = 0.994, *p* < 0.01; *β* = 1.006, *p* < 0.05). Income was significant only for the “frequent” participation category (*β* = 1.030, *p* < 0.01). Males reported higher sports participation than females. Better self-rated health was associated with higher sports participation (*β* = 0.818, *p* < 0.001; *β* = 0.825, *p* < 0.001; *β* = 0.826, *p* < 0.001). Residents in the East region showed higher sports participation than those in the West (*β* = 0.912, *p* < 0.001; *β* = 0.929, *p* < 0.001; *β* = 0.913, *p* < 0.001). Internet users reported significantly higher sports participation than non-users. Findings indicate that the family of origin’s social class positively moderates the association between education and sports participation. Individuals from higher social strata have greater opportunity and motivation to develop sports awareness through education than do disadvantaged groups, supporting Hypothesis 1b and rejecting Hypothesis 1a (Table 3 and Figure 1).

**Table 3:** Broadly ordered logit model for education, family-of-origin social class and sports participation (total sample) (*N* = 7,848).

Variable	*β*					
	*y* > 1	*y* > 2	*y* > 3	*y* > 1	*y* > 2	*y* > 3
Education	0.627***	0.688***	0.771***	0.673***	0.703***	0.747***
	(0.018)	(0.017)	(0.019)	(0.042)	(0.039)	(0.040)
Family-of-origin social class	0.921***	0.928***	0.949***	0.944**	0.934***	0.937**
	(0.012)	(0.012)	(0.012)	(0.026)	(0.024)	(0.024)
Education × Family-of-origin social class				0.983**	0.994**	1.006*
				(0.012)	(0.010)	(0.010)
Income	1.003	1.022	1.029**	1.003	1.022	1.030**
	(0.014)	(0.014)	(0.014)	(0.014)	(0.014)	(0.014)
Gender	0.875***	0.896**	0.921*	0.874***	0.895**	0.920*
	(0.045)	(0.043)	(0.044)	(0.045)	(0.043)	(0.044)
Political identity	0.750***	0.859*	0.825**	0.751***	0.858*	0.826**
	(0.070)	(0.070)	(0.064)	(0.070)	(0.070)	(0.064)
Health status	0.818***	0.822***	0.823***	0.818***	0.825***	0.826***
	(0.027)	(0.027)	(0.028)	(0.027)	(0.027)	(0.028)
Marital status	1.324***	1.224***	1.114***	1.325***	1.225***	1.110***
	(0.076)	(0.065)	(0.060)	(0.076)	(0.065)	(0.059)
Hukou	0.679***	0.659***	0.646***	0.680***	0.659***	0.645***
	(0.039)	(0.035)	(0.034)	(0.040)	(0.035)	(0.034)
Region	0.912***	0.929***	0.913***	0.912***	0.929***	0.913***
	(0.025)	(0.024)	(0.023)	(0.025)	(0.024)	(0.023)
Use Internet	0.648***	0.676***	0.673***	0.644***	0.677***	0.677***
	(0.043)	(0.045)	(0.046)	(0.043)	(0.045)	(0.046)
Age	1.005***	0.995**	0.983***	1.005***	0.995**	0.983***
	(0.002)	(0.002)	(0.002)	(0.002)	(0.002)	(0.002)
Constant	1.166***	1.958***	2.900***	1.061***	1.926***	2.961***
	(6.304)	(11.106)	(16.277)	(5.006)	(9.488)	(14.387)
Pseudo *R*^2^	0.0960	0.0960	0.0960	0.0962	0.0962	0.0962
Log-likelihood		−8883.13			−8870.14	
AIC		17838.25			17822.28	
BIC		18089.10			18050.03	

*p < 0.05, **p < 0.01, ***p < 0.001. Standard errors in parentheses.

Model comparison results show that, after adding the interaction term between education and family-of-origin social class, the log-likelihood increased from −8883.13 to −8870.14, while the AIC decreased from 17838.25 to 17822.28 and the BIC decreased from 18089.10 to 18050.03. Since lower AIC and BIC values indicate better model fit, these results suggest that the inclusion of the interaction term substantially improved model fit. Therefore, education and family-of-origin social class may have a significant moderating relationship.

### 4.3 Gender-stratified generalized ordered logit model results

Tables 4, 5 show gender differences in the association between education and sports participation. For men, a one-level increase in education raises the odds of sports participation by 2.007 [Exp(0.697)], 2.067 [Exp(0.726)], and 2.059 [Exp(0.722)] across the three cutpoints; for women, by 1.916 [Exp(0.650)], 1.975 [Exp(0.681)], and 2.164 [Exp(0.772)]. The effect of education thus differs by sex and participation level: among frequent participants, the effect is stronger for women; among infrequent (rarely/sometimes) participants, it is stronger for men. Higher family of origin social class does not uniformly boost men’s sports participation; its effect appears only at certain class thresholds. For men, higher family-of-origin social class improves “frequent” participation (*β* = 0.910, *p* < 0.01) and “sometimes” participation (*β* = 0.934, *p* < 0.05). For women, family of origin social class has a consistently positive effect on sports participation at all frequency levels (*β* = 0.952, *p* < 0.05; *β* = 0.926, *p* < 0.01; *β* = 0.965, *p* < 0.01). To examine the interaction between education and family-of-origin social class, an interaction term was added to the baseline model. The interaction model showed improved fit, with a higher log-likelihood (−4469.509 vs. −4450.12) and lower AIC (8939.018 vs. 8900.24) and BIC (8330.595 vs. 8305.80). We further conducted a formal likelihood-ratio test to compare the nested models. The result was statistically significant, LR *x*^2^ (1) = 38.778, *p* < 0.001, indicating that the inclusion of the interaction term significantly improved model fit.

**Table 4:** Broadly ordered logit model for education, family of origin social class and sports participation (male sample).

Variable	β					
	*y* > 1	*y* > 2	*y* > 3	*y* > 1	*y* > 2	*y* > 3
Education	0.653***	0.699***	0.766***	0.697***	0.726***	0.722***
	(0.028)	(0.026)	(0.028)	(0.063)	(0.058)	(0.055)
Family-of-origin social class	0.911***	0.924***	0.934***	0.932	0.934*	0.910**
	(0.018)	(0.018)	(0.018)	(0.039)	(0.376)	(0.036)
Education × Family-of-origin social class				0.985	0.991*	1.012*
				(0.018)	(0.015)	(0.014)
Income	1.064**	1.098***	1.116***	1.065**	1.099***	1.116***
	(0.028)	(0.027)	(0.026)	(0.028)	(0.027)	(0.026)
Political identity	0.669***	0.788**	0.744***	0.668***	0.785**	0.745***
	(0.076)	(0.080)	(0.073)	(0.076)	(0.080)	(0.073)
Health status	0.781***	0.797***	0.768***	0.780***	0.794***	0.773***
	(0.040)	(0.040)	(0.040)	(0.040)	(0.040)	(0.040)
Marital status	1.316***	1.251***	1.131**	1.327***	1.261***	1.117
	(0.116)	(0.102)	(0.082)	(0.117)	(0.103)	(0.091)
Hukou	0.710***	0.729***	0.709*	0.710***	0.730***	0.709***
	(0.063)	(0.059)	(0.056)	(0.063)	(0.059)	(0.056)
Region	0.900**	0.891***	0.929***	0.897**	0.888***	0.932*
	(0.037)	(0.034)	(0.035)	(0.037)	(0.034)	(0.035)
Use Internet	0.723***	0.761***	0.697***	0.718***	0.760***	0.704***
	(0.074)	(0.077)	(0.072)	(0.073)	(0.077)	(0.072)
Age	1.013***	1.004	0.987***	1.013***	1.004	0.987***
	(0.003)	(0.003)	(0.002)	(0.003)	(0.003)	(0.002)
Constant	0.345	1.078***	2.377***	0.239	1.015***	2.494***
	(1.194)	(4.001)	(8.979)	(0.722)	(3.267)	(8.113)
Pseudo *R*^2^	0.0983	0.0983	0.0983	0.0987	0.0987	0.0987
Log-likelihood		−4469.509			−4450.12	
AIC		8939.018			8900.24	
BIC		8330.595			8305.80	
Model comparison	LR *x*^2^	38.778	*p*-value	0.0008		

*p < 0.05, **p < 0.01, ***p < 0.001. Standard errors in parentheses.

**Table 5:** Broadly ordered logit model for education, family of origin social class and sports participation (female sample).

Variable	*β*					
	*y* > 1	*y* > 2	*y* > 3	*y* > 1	*y* > 2	*y* > 3
Education	0.600***	0.671***	0.767***	0.650***	0.681***	0.772***
	(0.024)	(0.024)	(0.027)	(0.057)	(0.052)	(0.058)
Family-of-origin social class	0.925***	0.920***	0.962**	0.952*	0.926**	0.965**
	(0.017)	(0.016)	(0.017)	(0.034)	(0.032)	(0.034)
Education × Family-of-origin social class				0.982**	0.996**	0.998**
				(0.017)	(0.014)	(0.014)
Income	0.980	0.994	0.989	0.980	0.994	0.989
	(0.017)	(0.016)	(0.017)	(0.017)	(0.016)	(0.017)
Political identity	0.882	0.898	0.923	0.882	0.900	0.925***
	(0.151)	(0.129)	(0.121)	(0.151)	(0.129)	(0.122)
Health status	0.844***	0.854***	0.861***	0.844***	0.854***	0.861
	(0.038)	(0.038)	(0.040)	(0.038)	(0.038)	(0.040)
Marital status	1.258***	1.110	1.003	1.257***	1.109	1.003***
	(0.098)	(0.081)	(0.073)	(0.097)	(0.081)	(0.073)
Hukou	0.656***	0.611***	0.598***	0.657***	0.611***	0.598***
	(0.052)	(0.045)	(0.044)	(0.052)	(0.045)	(0.044)
Region	0.923**	0.965	0.895***	0.924**	0.965	0.896***
	(0.035)	(0.034)	(0.032)	(0.035)	(0.034)	(0.032)
Use Internet	0.606***	0.630***	0.650***	0.604***	0.631***	0.651***
	(0.055)	(0.057)	(0.061)	(0.054)	(0.057)	(0.061)
Age	1.000	0.988***	0.979***	1.000	0.988***	0.979***
	(0.002)	(0.002)	(0.002)	(0.002)	(0.002)	(0.002)
Constant	1.608***	2.499***	3.275***	1.483***	2.475***	3.263***
	(6.327)	(10.229)	(13.189)	(5.135)	(8.873)	(11.495)
Pseudo *R*^2^	0.0997	0.0997	0.0997	0.0998	0.0998	0.0998
Log-likelihood		−5347.061			−5360.28	
AIC		9693.795			9682.56	
BIC		9903.701			9890.12	
Model comparison	LR *x*^2^	26.438	*p*-value	0.0007		

*p < 0.05, **p < 0.01, ***p < 0.001. Standard errors in parentheses.

There may be a significant moderating effect between education and family-of-origin social class. Education × family of origin social class was positively associated with men’s sports participation. A one-level increase in the interaction raised the odds of moving from “rarely” to “sometimes” by 2.693 [Exp(0.991)] (*p* < 0.05) and from “sometimes” to “frequent” by 2.752 [Exp(1.012)] (*p* < 0.05), indicating that men from higher-class families gain more achieving educational returns in the field of sports than men from lower-class families. Among women, family of origin social class showed a consistent moderating effect on the education–sports participation association: a one-level increase raised the odds of moving from “rarely” to “frequent” by 2.672 [Exp(0.982)], 2.708 [Exp(0.996)], and 2.712 [Exp(0.998)] (*p* < 0.01). The moderating effect of family of origin social class on the education–sports participation link thus differed by sex, with stronger statistical significance among women; in the full sample, increases in education enhanced sports participation more for women from lower-class families than for men. These findings support Hypothesis 2a and reject Hypothesis 2b. To test the interaction between education and family-of-origin social class, an interaction term was added to the baseline model. The interaction model showed better fit, with a higher log-likelihood (−5347.061 vs. −5360.28) and lower AIC (9693.795 vs. 9682.56) and BIC (9903.701 vs. 9890.12). These results suggest that education and family-of-origin social class may have a significant moderating relationship. We also conducted a formal likelihood-ratio test comparing the two nested models. The test was statistically significant, LR *x*^2^ (1) = 26.438, *p* < 0.001, indicating that adding the interaction term significantly improved model fit. Therefore, the results provide evidence of a moderating effect between education and family-of-origin social class.

### 4.4 Intergenerational effects of education and robustness checks

Education provides a basis for social mobility, which in turn contributes to a more equitable social structure and climate (22). As a robustness check, parental education was employed as an alternative proxy for family-of-origin social class to examine whether the main findings were sensitive to the measurement of family-of-origin social class (Table 6). For each one-level increase in father’s education, the odds of moving from “rarely” to “sometimes” and from “sometimes” to “frequently” were 2.693 [Exp(0.991)] (*p* < 0.01) and 2.450 [Exp(0.896)] (*p* < 0.001), respectively. Higher individual educational attainment was positively associated with sports participation (*β* = 0.534, *p* < 0.001; *β* = 0.610, *p* < 0.001; *β* = 0.713, *p* < 0.001). The interaction between individual education and father’s education was positive and statistically significant only among those who participated “frequently” (*β* = 1.040, *p* < 0.01). Mother’s education was also positively associated with offspring’s adult sports participation (*β* = 0.653, *p* < 0.001; *β* = 0.638, *p* < 0.001; *β* = 0.747, *p* < 0.01). In addition, the interaction between individual education and mother’s education was consistently positive and statistically significant across participation levels (*β* = 0.994, *p* < 0.05; *β* = 1.044, *p* < 0.01; *β* = 1.062, *p* < 0.01), suggesting that the beneficial association between individual education and sports participation was more evident when mothers’ education was higher. However, the Wald tests reported here assess the joint statistical significance of the maternal and paternal terms separately and do not constitute a direct test of whether the maternal coefficients are statistically larger than the paternal coefficients. The Wald test for maternal education and its interaction terms was statistically significant (Wald text = 25.36, *p* = 0.001), indicating a joint association between maternal education-related factors and offspring’s sports participation. In contrast, the corresponding Wald test for paternal education and its interaction terms was only marginally significant (Wald text = 8.72, *p* = 0.068). Therefore, rather than concluding that the maternal effect is definitively stronger than the paternal effect, these results suggest that maternal education shows more consistent and stronger statistical support in this robustness check.

**Table 6:** Broadly ordered Logit model for education, parental education and sports participation.

Variable	*β*					
	*y* > 1	*y* > 2	*y* > 3	*y* > 1	*y* > 2	*y* > 3
Education	0.534***	0.610***	0.713***	0.524***	0.593***	0.699***
	(0.025)	(0.024)	(0.025)	(0.030)	(0.026)	(0.027)
Father’s education	0.823	0.825**	0.896***			
	(0.074)	(0.063)	(0.060)			
Individual × Father’s education	0.966	1.007	1.040**			
	(0.027)	(0.022)	(0.020)			
Mother’s education				0.653***	0.638***	0.747**
				(0.096)	(0.079)	(0.085)
Individual × Mother’s education				0.994*	1.044**	1.062**
				(0.043)	(0.033)	(0.028)
Controls	Control variables	Control variables	Control variables	Control variables	Control variables	Control variables
Constant	0.982***	1.238***	1.332***	1.283***	1.476***	1.416***
	(8.464)	(11.473)	(12.569)	(7.650)	(10.222)	(10.437)
Pseudo *R*^2^	0.0708	0.0708	0.0708	0.0689	0.0689	0.0689
*z*-value	2.02	0.35	−1.19	2.23	1.34	−0.12
Wald text		8.72			25.36	
*p*-value		0.068			0.001	

*p < 0.05, **p < 0.01, ***p < 0.001. Standard errors in parentheses.

**Figure 2:**
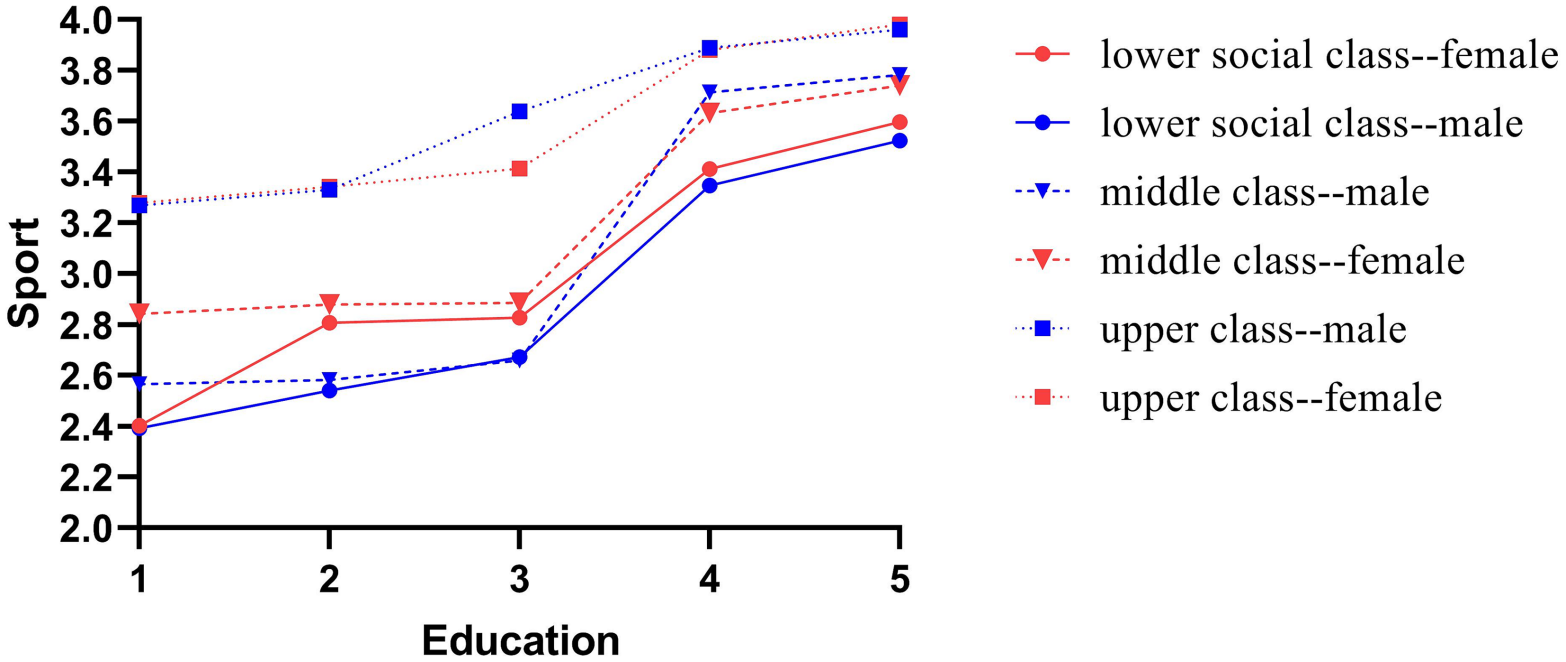
Marginal effect value chart.

The predicted values show a clear positive educational gradient across all gender and class groups. Individuals from higher social classes start with higher predicted values at the lowest level of education. However, the increase associated with education is larger among lower-class groups, suggesting that education may partly compensate for initial class disadvantages. Gender differences are relatively modest overall, although some differences appear within the middle-class group at higher levels of education (Figure 2).

## 5 Discussion

### 5.1 Chained relationship between social class, education, sports, and gender differences

The results show that family-of-origin social class and individual education are both independently related to sports participation. Higher educational attainment is associated with a greater likelihood of engaging in sport and may be linked to better access to sports-related information and sports resources, which together may help explain differences in sports participation behavior (23). Family-of-origin social class significantly moderates the association between education and sports participation: among individuals from higher-class backgrounds, the relationship between educational attainment and sports participation in adulthood is stronger. In the full sample, the findings are consistent with resource reinforcement theory: compared with individuals from disadvantaged backgrounds, those from advantaged backgrounds show a stronger positive association between education and sports participation, possibly reflecting their greater social and material resources.

Internet use has a significant positive association with sports participation in both the full sample and gender subsamples. When education is controlled, the magnitude of this association decreases but remains significant. Analyses of gender differences show that family-of-origin social class moderates the education–sports participation relationship more strongly and consistently among women, which is consistent with resource compensation theory (24). The findings suggest that education is importantly associated with patterns of sports participation among women from disadvantaged social origins. The consistently significant moderating role of family-of-origin social class across all frequency transitions indicates that the association between educational attainment and sports participation is not uniform across gender and class groups. In particular, higher educational attainment appears to be more strongly associated with sports participation among women from lower-class families than among men with similar social backgrounds. This pattern may reflect a compensatory process, whereby education is linked to alternative sources of empowerment when sports-related resources, opportunities, and health-oriented cultural capital are limited in the family of origin. Women from lower-class backgrounds with higher education may have greater health awareness and behavioral autonomy, as well as better access to social networks and institutional environments that are conducive to regular physical activity. In this sense, education may be associated with a partial reduction in the cumulative disadvantages related to both class and gender, with these women showing a stronger link between educational attainment and active lifestyles. By contrast, among women from higher-class families, education appears to be associated with the reinforcement of already existing advantages, although its marginal association may be less pronounced because of the relatively abundant resources available in their family-of-origin social class.

### 5.2 Intergenerational effects of class, education, and sports

This paper examines how parental education is associated with children’s sports participation through family climate and values and explores potential mediating factors in intergenerational transmission. Parental education is not only related to the reproduction of educational advantage but may also be linked to a supportive family environment for sports. Parental education is associated with children’s sports participation across generations, with maternal education showing consistently stronger associations than paternal education (25). These findings highlight the particularly important association between maternal education and offspring sports participation. Unlike paternal education, whose association appears to be more threshold-specific, maternal education was consistently associated with higher levels of sports participation across the full frequency spectrum. This pattern suggests that mothers’ educational attainment may be more stably and broadly linked to children’s physical activity behaviors. One possible explanation is that more educated mothers may be more likely to transmit health-related values, provide behavioral guidance, and create a family environment that is supportive of regular engagement in sports and physical activity. Moreover, the significant interaction effects indicate that maternal education is not only directly associated with sports participation but also related to stronger positive associations between individuals’ own education and sports participation. In this sense, maternal education may function as an intergenerational resource that is linked to a greater likelihood of education being associated with healthier and more active lifestyles. Children’s sports participation is related to the interplay between parental education and family resources. The association between family resources and sports participation appears to be stronger in families with more highly educated parents, indicating a pattern consistent with intergenerational reinforcement (26). This study traces the pathways through which parental resources may be associated with children’s sports participation. Systematic family support and a positive home environment may provide favorable conditions for sports engagement and are related to sustained participation in physical activity (27).

## 6 Conclusion and recommendations

The association between education and sports participation varies by sex: the positive association between higher education and sports participation is particularly pronounced among women from lower socioeconomic backgrounds. Regression models incorporating parental education as a key variable suggest the presence of intergenerational transmission. Parental education is indirectly associated with offspring’s sports participation through offspring’s own educational attainment, and the association with maternal education is stronger than that with paternal education. Higher educational achievement is positively associated with sports participation. The marginal association between education and sports participation appears to be stronger among those from lower socioeconomic backgrounds.

Overall, educational attainment is positively associated with sports participation, but this relationship varies by gender and family-of-origin social class. Education is related to higher levels of sports participation for both men and women, with stronger associations among men at lower participation levels and among women at higher participation levels. Family-of-origin social class shows a more consistent positive association with women’s participation, while its association with men’s participation appears only at specific thresholds. Interaction analyses further show that family-of-origin social class moderates the education–sports participation relationship. Higher family-of-origin social class is associated with a stronger education–sports participation relationship among men, whereas this moderating association is more stable and statistically robust among women. Improved model fit after adding the interaction term suggests that education and family-of-origin social class are jointly related to sports participation.

## 7 Limitations

This study has several limitations. First, because the analysis is based on cross-sectional data, the observed relationships should be interpreted as associations rather than causal effects. The study cannot establish temporal ordering or rule out reverse causality; therefore, no definitive conclusions can be drawn about whether family-of-origin social class or individual socioeconomic attainment causes differences in adult sports participation. Second, sports participation is measured using self-reported frequency, which may be affected by recall bias or social desirability bias. This measure also does not capture differences in the type, intensity, duration, or context of sports activities. Third, although the study controls for several relevant factors, unobserved variables such as health status, income, leisure time, access to sports facilities, and social support may still influence the results, leading to potential omitted-variable bias. Fourth, family-of-origin social class is a complex concept, and its measurement may not fully reflect all relevant dimensions, such as parental education, income, occupation, and cultural capital.

## Data Availability

The datasets presented in this study can be found in online repositories. The names of the repository/repositories and accession number(s) can be found in the article/Supplementary material.
